# Understanding the HIV-specific T-cell response to immune checkpoint blockade: what can we learn from cancer immunotherapy?

**DOI:** 10.1097/COH.0000000000000957

**Published:** 2025-07-18

**Authors:** Céline Gubser, Daniel E. Kaufmann

**Affiliations:** a Centre Hospitalier Univeristaire Vaudois, Epalinges; b Centre Hospitalier Univeristaire Vaudois, Lausanne, Switzerland

**Keywords:** functional HIV cure strategies, HIV-specific T-cells, immune checkpoint blockade, T-cell exhaustion and stemness

## Abstract

**Purpose of review:**

This review examines the potential of immune checkpoint blockade (ICB) to enhance HIV-specific T-cell responses, leveraging insights from cancer immunotherapy to tackle persistent challenges in achieving long-term potent immune response to keep the virus in check. By highlighting lessons from oncology, we aim to discuss innovative strategies to improve HIV treatment outcomes and advance the search for a functional cure.

**Recent findings:**

ICB extends beyond targeting PD-1 and CTLA-4, with novel therapies and engineered approaches in cancer also holding promise for HIV treatment. HIV-specific T-cell exhaustion, stemness, T-cell receptor clonal replacement, and antigen load critically influence ICB success, emphasizing the complexity and need for research on innovative strategies that can further enhance treatment efficacy in the context of HIV.

**Summary:**

While ICB shows promising potential, its role in HIV cure strategies requires further exploration in clinical trials with people with HIV (PWH). Future research should focus on advancing ICB as a tool for durable HIV control by investigating novel immune checkpoint targets, bispecific antibodies, minimizing toxicity, and identifying biomarkers for effective ICB responses.

## INTRODUCTION

T-cells are an essential part of the adaptive immune response against intracellular pathogens and cancer. They are both barrier and potential solution to an HIV cure. While CD4^+^ T-cells harbor the latent reservoir [1–3], CD8^+^ T-cells from HIV controllers, represent a key model for identifying factors contributing to long-term HIV remission [4^▪^]. Although antiretroviral therapy (ART) potently suppresses HIV replication and has dramatically improved prognosis for people with HIV (PWH), lifelong treatment remains necessary, immune dysfunction persists [5], and it does not provide a cure [6]. Despite significant scientific advances, to date, no functional or eradicating cure has been achieved. ICB and its dual role [7,8] of reactivating the virus [9,10] and reinvigorating the T-cell response [11–14] is actively investigated as a cure strategy for HIV with multiple ongoing clinical trials involving PWH both with and without cancer [15^▪^]. 

**Box 1:**
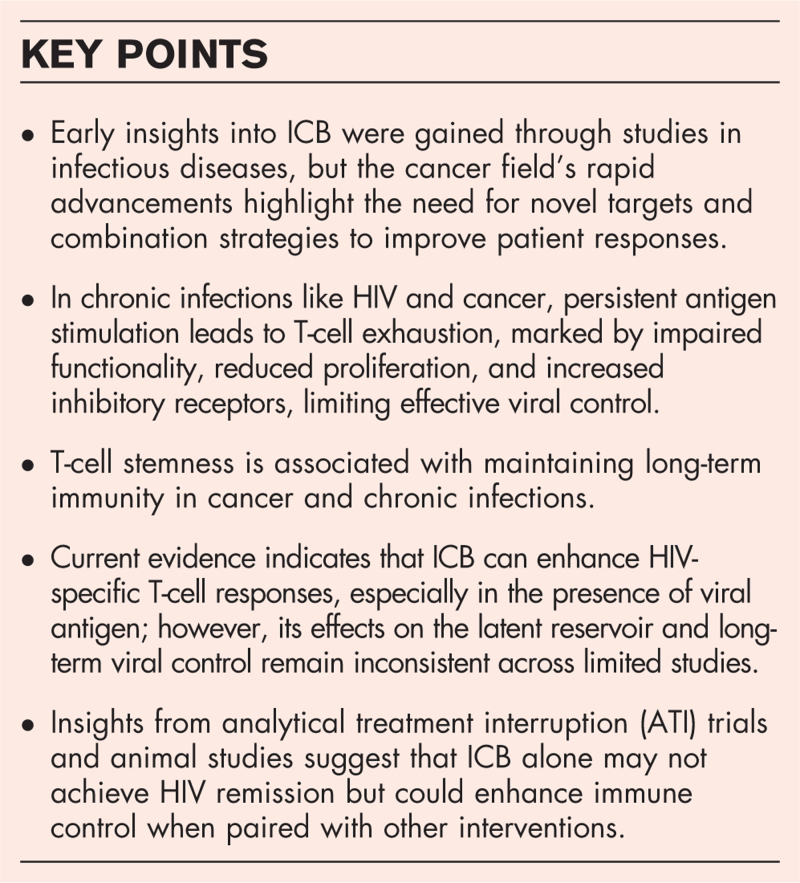
no caption available

## IMMUNE CHECKPOINT BLOCKADE AND ITS CONCEPTS

Early progress in understanding immune checkpoints and the potential of ICB was primarily made in infectious diseases through murine models and human observational studies. However, the cancer field has seen explosive progress [16–18] with knowledge gained in oncology now providing valuable insights for infectious diseases. Despite these advancements, patient responses remain inconsistent and often suboptimal [19^▪^]. The field is actively working on novel targets and combination strategies to overcome these limitations and expand the therapeutic reach of cancer immunotherapy.

### Blocking inhibitory receptors: releasing the brakes

**FIGURE 1:**
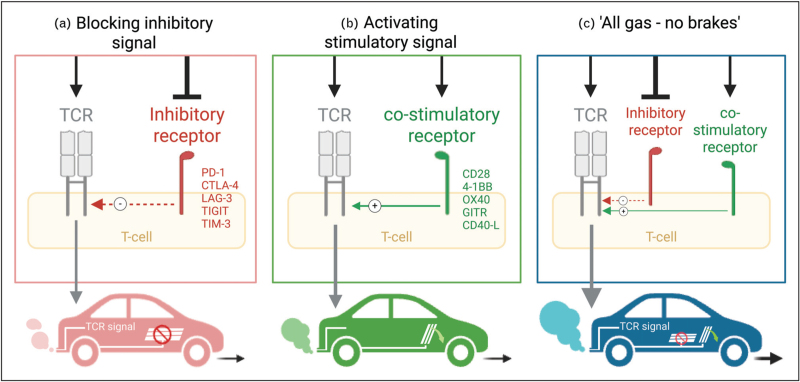
Immune checkpoint blockade concepts. (a) Blocking inhibitory receptors, such as PD-1 and CTLA-4, removes the negative regulation on T-cells, enhancing their activation by promoting the signaling through antigen: TCR interaction. (b) Activating co-stimulatory receptors such as CD28 and members of the TNFR superfamily enhances T-cell activation by providing a ”go" signal, thereby boosting the T-cell. (c) Finally, combining the blocking of inhibitory receptors with the stimulation of co-stimulatory receptors maximizes T-cell activation by removing inhibitory signals while enhancing co-stimulatory pathways, thus potentially creating a more potent and sustained immune response.

T-cells require two signals for full activation. One, via the antigen: T-cell receptor (TCR) binding and two, via a co-stimulatory signal, typically provided by CD28, which interacts with B7 molecules (CD80/CD86) on the antigen-presenting cells (APCs). Negative regulators such as PD-1 (programmed death-1), PD-L1/2 and CTLA-4 (cytotoxic T-lymphocyte associated protein 4) are inhibitory ICs that dampen proximal TCR signaling and compete for co-stimulatory binding. Targeting these ICs blocks the inhibitory receptor-ligand interaction and thereby takes the “breaks” off the activating TCR signals (Fig. 1a). While combination therapies with PD-1 and CTLA-4 result in enhanced T-cell activation in melanoma, it is also more toxic and carries an increased risk of immune-related adverse events (irAEs). A next wave of ICB targeting lymphocyte activation gene-3 (LAG-3), T-cell immunoglobulin and mucin-domain-containing-3 (TIM-3), and T-cell immunoreceptor with immunoglobulin and tyrosine-based inhibitory motif (ITIM) domain (TIGIT) has been well studied in clinical trials and hold promise of inducing less severe irAEs [20,21^▪^]. Recently, Opdualag, a fixed-dose combination of anti-LAG-3 and anti-PD-1, has shown significant therapeutic efficacy and was approved for treating melanoma in adults and children, marking LAG-3 as the third immune checkpoint pathway approved for clinical use by the FDA [22]. Ultimately, combining multiple ICB aims at optimizing treatment regimens, balancing efficacy with minimized risks and reduced resistance to single agent therapies.

### Activating co-stimulatory receptors: pressing the gas

A second approach is to actively enhance T-cell activation by stimulating co-stimulatory receptors like inducible co-stimulatory molecule (ICOS) or members of the TNF receptor (TNFR) superfamily like OX40 (CD134), 4–1BB (CD137), CD40L (CD154), glucocorticoid-induced TNFR-related protein (GITR), and CD27. Targeting these immune checkpoint further boost the T-cell response by providing an artificial “go” signal to the cells (Fig. 1b). From clinical trials in oncology, we know that many of these pathways show promising results but are either not efficacious when used as monotherapies [16,23,24] or too toxic. The CD28 super agonist antibody gained widespread attention due to participants experiencing cytokine storms and cardiovascular shock [25]. Nevertheless, new CD28-targeted approaches are emerging [26^▪^], as its critical role in maintaining long-term memory cells, supporting T-cell proliferation and survival during ICB has been well established [27,28]. Currently, a first-in-class CD28 bispecific antibody (REGN7075) is in clinical trials for colorectal cancer (NCT04626635) [29].

We recently showed that stimulating the co-stimulatory molecule GITR impaired rather than boosted the CD8^+^ HIV-specific immune response and we therefore concluded that GITR agonist antibodies should not be pursued for HIV cure strategies [30]. However, a recent study in a mouse tumor model found that intermittent administration of a MEK inhibitor combined with GITR and OX40 co-stimulation significantly enhanced T-cell function and decreased tumor growth [31^▪▪^], highlighting the complexity and context-dependency of co-stimulatory targeting.

### Combining blocking of inhibitory receptors with stimulation of co-stimulatory receptors: all gas, no brakes

A strategic approach that maximizes T-cell activation by simultaneously removing inhibitory signals and stimulating co-stimulatory pathways can create a more potent and sustained immune response (Fig. 1c). Targeting two immune checkpoint pathways using bispecific antibodies (bsAbs) is an exploding field in oncoengineering [32,33]. Leveraging their ability to temporally or spatially link two binding specificities is an advantage not achievable with conventional antibody combinations and opens new avenues for immunotherapy in cancer and chronic infections [34]. A growing number of anti-PD-L1-based bsAbs targeting both co-inhibitory and co-stimulatory molecules are being explored in biological testing and clinical trials (reviewed in Geng *et al.* 2024 [35^▪▪^]). Currently, a phase I clinical trial (NCT04762641) is assessing the safety of a PD-L1 x 4–1BB bsAb, which has demonstrated inhibition of tumor growth with increased CD8^+^ T-cell infiltration, and improved T-cell functionality with minimal toxicity in preclinical models of hepatocellular carcinoma and ovarian cancer [36^▪▪^]. Research on bsAbs in the HIV field has primarily focused on bridging immune cells (killers) with infected cells (targets) [37–39] and to date less on inducing “all gas, no brakes” of the exhausted immune response.

## HIV-SPECIFIC T-CELL DYSFUNCTION AND EXHAUSTION

Unlike acute infections, where antigen-specific T-cells expand, exert effector functions, and contract upon antigen clearance, persistent antigen stimulation in chronic infections like HIV or cancer induces alternative differentiation pathways that results in T-cell exhaustion [40]. This exhaustion is characterized by impaired functionality, reduced proliferative capacity, and upregulation of inhibitory receptors, limiting the immune system's ability to control the virus effectively [41].

Exhausted CD8^+^ T-cells have been well described in the context of chronic infections like HIV [42] and are characterized by upregulation of immune checkpoints such as PD-1, CTLA-4, TIM-3, LAG-3, GITR, and TIGIT, which suppress T-cell responses and cause loss of cytotoxic function [30,43–50]. They comprise a dynamic network with phenotypically and functionally distinct subpopulations [51,52]. The transcription factor thymocyte selection-associated HMG Box protein (TOX) has emerged as a critical master regulator driving T-cell exhaustion by orchestrating the exhaustion-specific transcriptional program and epigenetic landscape [53–55]. Metabolic impairment, including reprogramming and immunometabolism dysregulation, has been associated with loss of HIV control [56]. A recent study examining the transcriptional profile of HIV-specific T-cells in long-term nonprogressors (LTNPs) revealed that they exhibit increased protein/RNA metabolism, which may enhance T-cell functionality. It also suggests that energy-demanding RNA and protein metabolism contribute to CD8^+^ T-cell dysfunction in HIV progressors, potentially driven by mitochondrial dysfunction [57^▪▪^].

HIV-specific CD4^+^ T-cell dysfunction is also critical but less well described. Antigen-specific CD4^+^ T-cells play two crucial roles in chronic infections [58]: they provide help for the development of durable CD8^+^ T-cell memory [59] and are critically involved in the generation of neutralizing antibodies [60]. Early ART initiation has been shown to normalize T-cell activation [61] and help mitigate metabolic impairments and modulate PD-1 expression in CD4^+^ T-cells [62], preserving some immune functionality. While ART effectively suppresses viral replication, it does not fully restore T-cell homeostasis. PWH on ART show impaired T-helper cell responses compared to elite controllers, with deficits in Th1 and Th17/Th22 lineages that are crucial for antiviral immunity and mucosal defense [63]. This dysfunction may result, at least in part, to low-level viral antigen exposure during ART, which maintains elevated expression of immune checkpoints on HIV-specific CD4^+^ T-cells [64]. Their response to PD-L1 blockade *ex vivo* resulted in increased functionality only in certain T-helper subsets and suggests that the therapeutical potential of ICB may depend on the preexisting differentiation profile of CD4^+^ T-cells [43].

## T-CELL STEMNESS

Memory T-cells are quiescent, long-lived, and capable of retaining stemness if given sufficient time between challenges [65]. ICB research has provided crucial insights into the key role of stemness in sustaining long-term immunity in cancer and chronic infection [66]. In patients responding to PD-1 blockade therapy, the clinical benefit was attributed to a subpopulation of precursor exhausted T-cells (TPEX) CD8^+^ T-cells that exhibit both exhaustion markers and memory-stem-like characteristics [67–72]. These cells express the transcription factor T-cell-factor-1 (TCF-1) and were shown to undergo a proliferative burst upon PD-1 blockade, replenishing the exhausted CD8^+^ T-cell pool and enhancing immune responses against tumors [73,74]. A higher frequency of TPEX cells correlated with better antitumor responses and improved survival rates. The role of TCF-1 as regulator of stemness has also been described in a recent study using an algorithm to recover public single-cell transcriptomic datasets from seven human cancers highlighting the importance of a TCF1-CXCR6-axis regulating T-cell dysfunction and antitumor immunity [75]. In murine models, CXCR6 deletion dampened CD28 signaling and impaired tumor control by increasing apoptosis in effector PD-1+TIM3+ T-cells [75]. CD28 plays a critical role in maintaining TPEX cells during ICB [28,76]. These studies underscore the importance of TCF-1 in balancing T-cell activation and persistence in chronic immune responses [75]. A recent study by Utzschneider *et al.*[77^▪▪^] demonstrated that the transcriptional regulator inhibitor of DNA binding 3 (ID3) marks a subset of stem-like antigen experienced CD8^+^ memory T-cells that were uniquely adapted to generate progeny capable of sustaining immune responses during chronic infections in a mouse model of lymphocytic choriomeningitis virus (LCMV). The IL-1 cytokine family was crucial for expansion of ID3+ precursors, and their loss resulted in impaired immune control [77^▪▪^].

In SIV infection of nonhuman primates, a TOXhiTCF1+CD39+ memory subset with simultaneous effector and stem-like profiles was associated with limited viral persistence [78^▪▪^]. This subset of antigen-responsive SIV-specific CD8^+^ T-cells exhibited high inhibitory receptor (PD-1, TGIT, LAG-3) expression but retained stem-like properties, forming a lineage relationship with terminal effector T-cells identified by the same TCR clonality. PD-1 blockade resulted in a selective proliferative increase of CD8^+^ effector T-cells, further highlighting their progenitor-progeny relationship [78^▪▪^]. Higher expression of TCF-1 was also shown in CD8^+^ T-cells of HIV elite controllers compared to noncontrollers [79]. A recent study established a three-phase model (priming-expanding-rejuvenating) for inducing long-term viral control in SIV-infected macaques by combining IL-10 and PD-1 blockade, demonstrating that nine out of ten animals achieved significantly lower viremia levels after ART interruption (ATI) compared to control groups [80^▪▪^]. The researchers observed that lower viral levels correlated with higher frequencies of memory T-cells expressing TCF-1 and increased SIV-specific CD4^+^ and CD8^+^ T-cells in both blood and lymph nodes 24 weeks post-ATI [80^▪▪^]. The identification of stem-like CD8^+^ T-cells in both cancer and HIV suggests that enhancing T-cell stemness in future immunotherapies could improve immune-mediated control over HIV.

## T-CELL RECEPTOR CLONAL REPLACEMENT

Recent cancer studies suggest that effective ICB depends not just on reinvigorating exhausted T-cells but also on recruiting new functional T-cell clones [81]. This process, known as T-cell clonal replacement or de-novo priming, involves recruitment of naive or memory T-cells into the tumor microenvironment, expansion of new T-cell clones that recognize tumor antigens [82] and replacement of exhausted clones with more functional, proliferative T-cells capable of sustained tumor control. A study in site-matched tumors from patients with basal or squamous cell carcinoma receiving anti-PD-1 therapy found that posttherapy exhausted CD8^+^ T-cells were predominantly composed of novel TCR clonotypes, indicating a process of clonal replacement, where the immune response to immunotherapy is driven by newly expanded TCR clonotypes (potentially with new antigen specificities) rather than reinvigoration of preexisting clones [83]. While HIV-1-infected individuals on ART show quantitative but not qualitative impairment in naive CD8^+^ T-cell priming [84], ICB was shown to lower the threshold for naive T-cell priming [85]. HIV-specific T-cell frequencies correlates with viral RNA levels during ART [64,86], suggesting that low-level antigen maintains this pool of cells. If ICB were to work through the dual mechanism of reinvigorating exhausted CD8^+^ T-cells and promoting expansion of newly primed T-cells, a polyclonal, diverse HIV-specific TCR repertoire could improve viral control after ART cessation. The HIV TCR repertoire was recently shown to undergo rejuvenation under long-term ART. Analysis in PWH over a period of 25 years showed “clonal succession” of newly dominant CD8^+^ TCR clonotypes that exhibited stem-like profiles [87^▪▪^]. However, this was in the absence of ICB.

## ROLE FOR ANTIGEN

Tumor studies suggest that antigen processing and presentation pathways significantly influence ICB outcomes, as tumors with poor antigen expression typically demonstrate limited T-cell infiltration and minimal ICB response [88].

Studies in SIV infected nonhuman primates show that blocking anti-PD-L1 has minimal effect when given during suppressive ART [89]. Dual blockade of PD-1 and CTLA-4 showed enhanced viral reactivity and reduction of total levels of integrated virus but failed to control viral rebound upon analytical treatment interruption (ATI) [90]. The study concluded that ICB targeting PD-1 and/or CTLA-4 are unlikely to induce HIV remission in PWH in the absence of additional interventions. However, timing of ICB relative to ART and ATI appears to play a key role for its impact. Administering anti-PD-1 before starting ART and again after ART improved outcomes, including faster viral suppression and enhanced T-cell function [91]. Similarly, giving anti-PD-1 when stopping ART improved CD8^+^ T-cell function and viral control [92,93] and transiently increased functional SIV-specific CD8^+^ T-cells. These findings suggest ICB's effectiveness increases with viral antigen presence.

Data from clinical investigations in PWH suggest targeting the PD-1 pathway may increase HIV-specific T-cell responses [94], but effects on the HIV reservoir remain inconsistent [10,95–97]. An early study of a single low-dose anti-PD-L1 antibody (BMS-936559) in PWH on ART significantly enhanced of HIV-specific cytokine-producing T-cells in two out of six participants [98]. Comparable immunological improvements were observed in one out of four participants during a phase 1 trial evaluating an anti-PD-1 antibody [99^▪^]. The impact of combination therapy with anti-PD-1 and anti-CTLA-4 in a clinical study of PWH on ART and cancer resulted in a modest increase in cell-associated unspliced HIV RNA, with no overall change in replication-competent HIV, although a decrease in inducible virus was observed in two participants using the quantitative viral outgrowth assay [97].

Given that PWH on ART have minimal circulating viral antigen [64,100,101], larger changes might occur if ICB were given during treatment interruption rather than during stable ART. Indeed, a clinical trial evaluating the PD-1 inhibitor budigalimab (NCT 04223804) in PWH assessed its effect on viral rebound during an ATI [102]. Results showed no significant impact on viral dynamics when given before ATI, however, when administered during ATI, six out of nine participants exhibited some level of post-ART viral control, including two with strict postintervention suppression (<200 copies/ml) for up to 29 weeks.

Cancer vaccines using tumor-associated antigens in combination with ICB were shown to create a synergistic effect by expanding the pool of tumor-specific T-cells and enhancing their functionality [103^▪^]. In animal models of HIV, enhanced immune responses have been observed when ICB is combined with both prophylactic and therapeutic vaccinations [104–108]. Future clinical trials are necessary to test if therapeutic T-cell based vaccines in combination with ICB can elicit a long-lived, highly functional HIV-specific immune response and serve as an HIV cure strategy.

## CONCLUDING REMARKS

While ICB has demonstrated success in reinvigorating exhausted T-cells in cancer, its potential as an adjuvant strategy in HIV therapy remains to be better defined. Current evidence suggests that ICB can enhance HIV-specific T-cell responses, particularly in the presence of viral antigen, but based on limited studies its effects on the latent reservoir and long-term viral control are inconsistent. Insights from ATI trials and monkey studies suggest that ICB alone is unlikely to achieve HIV remission but may contribute to improved immune control when combined with other interventions. The emerging insights into T-cell stemness, clonal replacement and antigen load in immune responses opens new avenues for optimizing ICB strategies.

While ICB was historically limited in PWH due to concerns about viral reactivation and irAEs, recent data indicate that ICB is well tolerated in this population, with irAE rates similar to those observed in HIV-negative individuals [109,110,111^▪▪^]. Although established management protocols exist for irAEs in cancer, the tolerance for these events is lower in otherwise healthy PWH, necessitating strategies such as lower doses and less frequent ICB administration, which have shown promise in reducing irAEs [112,113] and are currently being tested in the NIVO-LD study (NCT05187429) for PWH on ART receiving low-dose nivolumab, an anti-PD-1 antibody. An exciting ongoing phase 1 clinical trial in PWH on ART and refractory Kaposi Sarcoma, NCT05646082 aims to evaluate the safety of dostarlimab, a PD-1 blocking monoclonal antibody. Dostarlimab recently showed unprecedent success, with sustained, clinical complete response rates in 41 of 47 patients with rectal cancer [114^▪▪^]. Future research should explore bispecific antibodies, integrating immune modulators to poise memory formation, and identifying biomarkers to predict ICB responders, ultimately advancing ICB as a strategy for a functional HIV cure.

## Acknowledgements

*None.*

### Financial support and sponsorship

*None.*

### Conflicts of interest

*There are no conflicts of interest.*

## REFERENCES AND RECOMMENDED READING

Papers of particular interest, published within the annual period of review, have been highlighted as:

▪ of special interest
▪▪ of outstanding interest
